# Falls Predict Fractures Independently of FRAX Probability: A Meta‐Analysis of the Osteoporotic Fractures in Men (MrOS) Study

**DOI:** 10.1002/jbmr.3331

**Published:** 2017-12-08

**Authors:** Nicholas C Harvey, Anders Odén, Eric Orwoll, Jodi Lapidus, Timothy Kwok, Magnus K Karlsson, Björn E Rosengren, Östen Ljunggren, Cyrus Cooper, Eugene McCloskey, John A Kanis, Claes Ohlsson, Dan Mellström, Helena Johansson

**Affiliations:** ^1^ MRC Lifecourse Epidemiology Unit University of Southampton Southampton UK; ^2^ NIHR Southampton Biomedical Research Centre University of Southampton and University Hospital Southampton NHS Foundation Trust Southampton UK; ^3^ Centre for Bone and Arthritis Research (CBAR), Sahlgrenska Academy University of Gothenburg Gothenburg Sweden; ^4^ Centre for Metabolic Bone Diseases University of Sheffield Sheffield UK; ^5^ Oregon Health and Science University Portland OR USA; ^6^ Department of Public Health and Preventive Medicine, Division of Biostatistics Oregon Health and Science University Portland OR USA; ^7^ Department of Medicine and Therapeutics and School of Public Health The Chinese University of Hong Kong Hong Kong; ^8^ Clinical and Molecular Osteoporosis Research Unit, Department of Clinical Sciences Malmo Lund University and Department of Orthopedics, Skane University Hospital Malmo Sweden; ^9^ Department of Medical Sciences University of Uppsala Uppsala Sweden; ^10^ NIHR Oxford Biomedical Research Centre University of Oxford Oxford UK; ^11^ Centre for Integrated Research in Musculoskeletal Ageing (CIMA), Mellanby Centre for Bone Research University of Sheffield Sheffield UK; ^12^ Institute for Health and Aging Catholic University of Australia Melbourne Australia

**Keywords:** OSTEOPOROSIS, EPIDEMIOLOGY, FRAX, FALLS, FRACTURE, INTERACTION

## Abstract

Although prior falls are a well‐established predictor of future fracture, there is currently limited evidence regarding the specific value of falls history in fracture risk assessment relative to that of other clinical risk factors and bone mineral density (BMD) measurement. We therefore investigated, across the three Osteoporotic Fractures in Men (MrOS) Study cohorts, whether past falls predicted future fracture independently of FRAX and whether these associations varied with age and follow‐up time. Elderly men were recruited from MrOS Sweden, Hong Kong, and USA. Baseline data included falls history (over the preceding 12 months), clinical risk factors, BMD at femoral neck, and calculated FRAX probabilities. An extension of Poisson regression was used to investigate the associations between falls, FRAX probability, and incident fracture, adjusting for age, time since baseline, and cohort in base models; further models were used to investigate interactions with age and follow‐up time. Random‐effects meta‐analysis was used to synthesize the individual country associations. Information on falls and FRAX probability was available for 4365 men in USA (mean age 73.5 years; mean follow‐up 10.8 years), 1823 men in Sweden (mean age 75.4 years; mean follow‐up 8.7 years), and 1669 men in Hong Kong (mean age 72.4 years; mean follow‐up 9.8 years). Rates of past falls were similar at 20%, 16%, and 15%, respectively. Across all cohorts, past falls predicted incident fracture at any site (hazard ratio [HR] = 1.69; 95% confidence interval [CI] 1.49, 1.90), major osteoporotic fracture (MOF) (HR = 1.56; 95% CI 1.33, 1.83), and hip fracture (HR = 1.61; 95% CI 1.27, 2.05). Relationships between past falls and incident fracture remained robust after adjustment for FRAX probability: adjusted HR (95% CI) any fracture: 1.63 (1.45, 1.83); MOF: 1.51 (1.32, 1.73); and hip: 1.54 (1.21, 1.95). In conclusion, past falls predicted incident fracture independently of FRAX probability, confirming the potential value of falls history in fracture risk assessment. © 2017 The Authors. *Journal of Bone and Mineral Research* Published by Wiley Periodicals Inc.

## Introduction

Although low bone mineral density is a major risk factor for fragility fracture, the majority of such low‐trauma fracture events occur as a result of a fall from standing height or less.^1^ Conversely, the number of falls is much greater than the number of consequent fractures with only 5% to 10% of falls in older adults leading to skeletal injury.^1^ Interventions aimed at reducing falls have usually been unsuccessful at reducing fractures,^2^, ^3^ probably partly as a consequence of the low falls to injury ratio. Notwithstanding, prior falls have been found to be a risk factor for future fracture in a number of cohorts.^4^ With the advent of the FRAX fracture risk assessment tool, evaluation of an individual's probability of sustaining a hip or major osteoporotic fracture over a 10‐year time period is now readily undertaken using a small number of easily ascertainable clinical risk factors and BMD if available.^5^ FRAX is the most widely used fracture risk assessment tool, incorporated into the majority of assessment guidelines worldwide^6^ but, unlike other tools such as QFracture or the GARVAN calculator,^7^, ^8^, ^9^ does not include falls as a specific input risk factor^4^, ^5^ because of the inconsistent data across the 12 derivation and 11 validation cohorts.^10^ In order for prior falls to be useful in the current context of risk assessment, the associated fracture risk must ideally be independent of FRAX probability and/or BMD. Having demonstrated that the risk of future falls associated with past falls is partly captured by FRAX,^11^ we undertook to investigate, across the three Osteoporotic Fractures in Men (MrOS) Study cohorts, whether a history of past falls (in the previous 12 months) independently predicted future fractures and whether the predictive value varied with follow‐up time or age.

## Subjects and Methods

### Participants

Details of the Osteoporotic Fractures in Men (MrOS) International Study have been published previously,^12^, ^13^ but briefly, MrOS is a multicenter study of community‐dwelling men aged 65 years or older from three countries, recruited and evaluated using similar criteria. To be eligible for the study, subjects had to be able to walk without aid. In the MrOS Hong Kong Study, 2000 Chinese men, aged 65 to 92 years, were enrolled between August 2001 and February 2003.^14^ All were Hong Kong residents of Asian ethnicity. Stratified sampling was adopted to ensure that 33% of subjects were included in each of the following age groups: 65 to 69, 70 to 74, and ≥75 years. Recruitment notices were placed in housing estates and community centers for the elderly. In the MrOS Sweden Study, 3014 men, aged 69 to 81 years, were enrolled between October 2001 and December 2004.^11^, ^15^ The cohort comprised men from the cities of Malmo, Gothenburg, and Uppsala, identified and recruited using national population registers. More than 99% were of white ethnicity. The participation rate in the MrOs Sweden Study was 45%. In the MrOS United States Study, 5995 men, aged 65 to 100 years, were enrolled at six sites between March 2000 and April 2002.^16^, ^17^ Each US clinical site designed and customized strategies to enhance recruitment of its population. Common strategies included mailings from the Department of Motor Vehicles, voter registration and participant databases, common senior newspaper features and advertisement, and targeted presentations. Self‐defined racial/ethnic ancestry was ascertained through questionnaires at baseline.

### Exposure variables

At baseline, height (centimeters) and weight (kilograms) were measured, and body mass index (BMI) was calculated as kilograms per square meter. The international MrOS questionnaire^16^ was administered at baseline to collect information about current smoking, number and type of medications, fracture history, family history of hip fracture, past medical history (rheumatoid arthritis), and high consumption of alcohol (3 or more glasses of alcohol‐containing drinks per day), calculated from the reported frequency and amount of alcohol use. Previous fracture at baseline was documented as all fractures after the age of 50 years, regardless of trauma. For glucocorticoid exposure, this was documented in MrOs as use at least 3 times per week in the month preceding the baseline assessment. Apart from rheumatoid arthritis, there was no information on secondary causes of osteoporosis, and the input variable for FRAX probability calculation was set to no for all men. Self‐reported falls during the 12 months preceding the baseline were recorded by questionnaire (past falls). Areal bone mineral density (aBMD) was measured at the femoral neck (FN) using Hologic QDR 4500 A or W (Hologic, Bedford, MA, USA) or Lunar Prodigy (GE Lunar Corp., Madison, WI, USA) depending on the center, with cross calibration of instruments. A *T*‐score was calculated using NHANES young women as a reference value.^18^ Ten‐year probability of fracture (FRAX major osteoporotic fracture [hip, humerus, vertebral, or forearm sites]) was calculated using clinical risk factors described above with and without femoral neck BMD entered into country‐specific FRAX models. Because the gradients of risk for incident falls were similar with either model, results for the models including femoral neck BMD are presented.

### Fracture and death outcomes

#### Hong Kong^19^

Incident fractures were captured via subject follow‐up through phone call or visit to the research center. All fracture sites (hip, wrist, skull/face, ribs, shoulder, arm, wrist, vertebra, tibia, fibula, foot, metatarsal toes, hand, fingers, and pelvis) were recorded. Pathological fractures were excluded. All incident fractures reported by participants were then confirmed by radiograph or medical record. Deaths were verified by death certificates.

#### Sweden^20^

Central registers covering all Swedish citizens were used to identify the subjects and the time of death for all subjects who died during the study, and these analyses were performed after the time of fracture validation. At the time of fracture evaluation, the computerized X‐ray archives in Malmo, Gothenberg, and Uppsala were searched for new fractures occurring after the baseline visit using the unique personal registration number allocated to every Swedish citizen. All additional fractures reported by the study subject after the baseline visit were confirmed by physician review of radiology reports. Fractures reported by the study subject but not possible to confirm by radiographic report were not included.

#### US^16^

If a participant reported a fracture, study staff conducted a follow‐up telephone interview to determine the date and time the fracture occurred, a description of how the fracture occurred, the type of trauma that resulted in the fracture, the participant's location and activities at the time of the fracture, symptoms just before or coincident with the fracture, and source of medical care for the fracture. All reported fractures were verified by a physician adjudicator through medical records obtained from the participant's physician. The Clinical Outcomes Committee adjudicated any uncertainties regarding the presence of a fracture. Deaths were verified through state death certificates.

### Statistical methods

To compare the performance of FRAX probability with that of a history of past falls, a dichotomous variable was created such that the percentage of men who had a high fracture risk was similar to the percentage who had previously fallen (15% for HK, 16% for Sweden, and 20% for US). Thus, 15%, 16%, and 20% men, respectively, had a FRAX probability of major osteoporotic fracture, calculated with BMD, above 9.5%, 15.8%, and 10.3% and the dichotomized FRAX score was therefore classified as high or low risk. Fracture outcomes considered included: any, osteoporotic (defined according to Kanis and colleagues^21^ as clinical vertebral, ribs, pelvis, humerus, clavicle, scapula, sternum, hip, other femoral fractures, tibia, fibula, distal forearm/ wrist), major osteoporotic (MOF; hip, clinical vertebral, humerus, and wrist/forearm), osteoporotic fracture without hip fracture (clinical vertebral, humerus, and wrist), clinical vertebral, and hip. An extension of Poisson regression models^22^ was used to study the association between FRAX, other risk variables, and the future risk of fracture. All associations were adjusted for age and time since baseline. In contrast to logistic regression, the Poisson regression uses the length of each individual's follow‐up period, and the hazard function is assumed to be exp(β_0_ + β_1 _× current time from baseline + β_2 _× current age + β_3 _× variable of interest). The observation period of each participant was divided into intervals of 1 month. One fracture per person, and time to the first fracture, were counted, and time at risk was censored at the time of first fracture, migration, or death. Thus, we investigated the predictive value of prior falls, FRAX (including each individual constituent risk factor), and BMD as individual risk factors, and then in multivariable models to investigate the value of falls independent of FRAX or BMD, and FRAX independent of falls. In further analyses, we explored interactions with age and time since baseline, in which age and time were used as continuous variables and examples given at specific ages and times. Additionally, we stratified the analyses by femoral neck BMD *T*‐score above or below –2.5. The association between predictive factors and risk of fracture are described as a hazard ratio (HR) or gradient of risk (GR = HR per 1 standard deviation change in predictor in the direction of increased risk). In addition, we explored the associations between falls and fracture by number of falls reported at baseline (1 versus multiple). Two‐sided *p* values were used for all analyses, and *p* < 0.05 was considered to be significant. Analyses were undertaken separately within each cohort and then the β‐coefficients from each cohort were weighted according to the variance and merged to determine the weighted mean of the coefficient and its standard deviation (random‐effects meta‐analysis). The risk ratios are then given by e^(weighted mean coefficient)^. Although there are numerous caveats with the use of receiver operator curve (ROC) models in this context,^23^ we additionally present area under the curve (AUC) values for the predictive models in the Supplemental Tables.

## Results

### Characteristics of participants

The study cohort consisted of 7857 men who had information on falls, BMD, and FRAX risk factors: 4365 men in the US (mean age 73.5 years; mean follow‐up 10.8 years); 1823 men in Sweden (mean age 75.4 years; mean follow‐up 8.7 years); and 1669 men in Hong Kong (mean age 72.4 years; mean follow‐up 9.8 years). Table 1 summarizes the baseline characteristics of the individuals by country cohort. Rates of past falls were similar at 20%, 16%, and 15% respectively. Rates of previous fracture were higher in Sweden (33%) than in the US (22%) and Hong Kong (13%). Consistent with the known country‐specific epidemiology of fracture, the highest mean FRAX probability was observed in Sweden (11.4% probability of major osteoporotic fracture, calculated with BMD), followed by the US (7.9%) and Hong Kong (6.7%).

**Table 1:** Baseline Characteristics of MrOS Participants by Country Cohort

	Hong Kong	Sweden	USA
Proportion of whole cohort	83%	61%	73%
No. of participants	1669	1823	4365
Person‐years	16,423	15,878	47,044
Age (years), mean (range)	72.4 (65–91)	75.4 (70–81)	73.5 (64–99)
Body mass index	23.5 ± 3.2	26.3 ± 3.6	27.42 ± 3.9
Previous fracture	13%	33%	22%
Family history hip fracture	5%	13%	17%
Smoker	12%	8%	3%
Steroid	1%	2%	2%
Rheumatoid arthritis	1%	1%	5%
Excess alcohol	1%	3%	4%
BMD FN *T*‐score	–1.4 ± 0.9	–0.9 ± 1.0	–0.6 ± 1.1
Fall at baseline	15%	16%	20%
No. falls at baseline			
0: 0 times	1426 (85%)	1538 (84%)	3478 (80%)
1: 1 time	192 (12%)	162 (9%)	519 (12%)
2: 2–3 times	42 (3%)	85 (5%)	305 (7%)
3: 4–5 times	6 (0.4%)	14 (0.8%)	41 (0.9%)
4: 6+ times	3 (0.2%)	13 (0.7%)	22 (0.5%)
FRAX MOF without BMD (mean ± SD)	6.9 ± 2.9	13.5 ± 6.2	9.2 ± 5.0
FRAX hip without BMD (mean ± SD)	3.4 ± 2.6	7.5 ± 5.5	3.7 ± 4.0
FRAX MOF with BMD (mean ± SD)	6.7 ± 3.3	11.4 ± 6.8	7.9 ± 4.8
FRAX hip with BMD (mean ± SD)	3.1 ± 2.7	5.6 ± 6.1	2.5 ± 3.6
High FRAX (ost with BMD)	15%	16%	20%
Threshold for high FRAX (%)	9.50	14.00	10.30
FU (hip fx: mean (SD), years)	9.8 (2.9)	8.7 (2.8)	10.8 (3.8)
Any fx	11%	23%	19%
Osteoporotic fx	9%	19%	14%
MOF fx	7%	16%	10%
Hip fx	3%	7%	4%

BMD = bone mineral density; FN = femoral neck; Fx = fracture; Ost = osteoporotic; MOF = major osteoporotic fracture.

### Past falls, FRAX probability, and risk of incident fracture

Table 2 summarizes the relationships between past falls or high FRAX probability at baseline and incident fractures. Supplemental Table S1 additionally presents the predictive value of the individual FRAX risk factors and of falls adjusted for each of these variables. Across all cohorts, past falls predicted any incident fracture (HR = 1.69; 95% CI 1.49, 1.90), major osteoporotic fracture (HR = 1.56; 95% CI 1.33, 1.83), and hip fracture (HR = 1.61; 95% CI 1.27, 2.05). Similar relationships were found for osteoporotic fracture and major osteoporotic fracture without hip fracture, summarized in Table 2. The predictive value of past falls was present within each individual cohort apart from when hip fracture was the outcome, where for Sweden and Hong Kong, the 95% CI included unity. The magnitudes of the gradients of risk were similar in Sweden and US cohorts, and marginally higher in the Hong Kong cohort, albeit with substantially overlapping confidence intervals, and there was thus no statistically significant interaction between falls and center. For illustrative purposes, Supplemental Table S2 demonstrates the fracture incidence amongst the four groups defined by high versus low FRAX probability and falls yes/no; the hazard ratio for major osteoporotic fracture is also given for the remaining three groups relative to the low FRAX probability and no falls groups.

**Table 2:** Relationships Between Past Falls, FRAX, and Risk of New Fracture

		Any fx	Ost fx	MOF	Hip fx
Falls at baseline	HK	1.93 (1.38, 2.70)	1.83 (1.25, 2.68)	2.01 (1.32, 3.05)	1.71 (0.92, 3.21)
	SW	1.61 (1.27, 2.03)	1.50 (1.16, 1.94)	1.50 (1.13, 1.98)	1.34 (0.85, 2.09)
	US	1.67 (1.43, 1.94)	1.54 (1.29, 1.84)	1.50 (1.21, 1.86)	1.74 (1.27, 2.38)
	Total	1.69 (1.49, 1.90)	1.56 (1.36, 1.79)	1.56 (1.33, 1.83)	1.61 (1.27, 2.05)
High FRAX (MOF with BMD)	HK	2.45 (1.78, 3.38)	3.04 (2.14, 4.32)	3.20 (2.17, 4.72)	5.27 (3.07, 9.05)
	SW	1.76 (1.40, 2.21)	1.83 (1.43, 2.34)	1.98 (1.52, 2.57)	1.82 (1.21, 2.74)
	US	2.01 (1.74, 2.33)	2.13 (1.80, 2.52)	2.29 (1.87, 2.79)	2.84 (2.11, 3.81)
	Total	2.00 (1.73, 2.31)	2.21 (1.75, 2.79)	2.35 (1.87, 2.94)	2.93 (1.75, 4.88)

Fx = fracture; Ost = osteoporotic; MOF = major osteoporotic fracture; HK = Hong Kong; SW = Sweden; US = United States; BMD = bone mineral density.

Data are hazard ratios (95% CI) adjusted for age and time since baseline.

The hazard ratio associated with multiple falls tended to be marginally greater than that associated with a single fall, for example, HR for 1 fall for MOF = 1.56 (95% CI 1.33, 1.83) and HR for ≥2 falls = 2.00 (95% CI 1.35, 2.98). High FRAX probability of major osteoporotic fracture, calculated with BMD, was predictive of all fracture outcomes, with the magnitude of the HR greater than for the equivalent falls‐fracture relationships (summarized in Table 2). Thus, across all cohorts, high FRAX predicted any incident fracture (HR = 2.00; 95% CI 1.73, 2.31), major osteoporotic fracture (HR = 2.35; 95% CI 1.87, 2.94), and hip fracture (HR = 2.93; 95% CI 1.75, 4.88).

### Independent predictive value of falls, FRAX probability, and BMD

The relationships between past falls and incident fracture remained robust after adjustment for high FRAX probability (MOF): adjusted HR (95% CI) any fracture: 1.63 (1.45, 1.83); MOF: 1.51 (1.29, 1.77); and hip: 1.54 (1.21, 1.95), and for BMD (Table 3). Indeed, the hazard ratios and 95% CI were very little altered by adjustment for high FRAX probability or BMD. Similarly, the gradient of risk for fracture outcomes with high FRAX probability were little altered by adjustment for the presence of reported past falls at baseline (Table 3): adjusted HR (95% CI) any fracture: 1.96 (1.69, 2.27); MOF: 2.30 (1.84, 2.88); and hip: 2.86 (1.73, 4.75). The associations with the outcomes of clinical vertebral fracture and osteoporotic fracture without hip fracture (OWH) are documented in Supplemental Table S3, demonstrating that both prior falls and FRAX probability were predictive of both outcomes. Supplemental Table S4 demonstrates the predictive value of these exposures when femoral neck BMD at baseline is dichotomized above/below *T *= –2.5. For illustrative purposes, the AUC values for the different prediction models are presented in Supplemental Table S5.

**Table 3:** Past Falls Adjusted for FRAX Probability and FRAX Probability Adjusted for Past Falls, as Predictors of Incidence Fracture

		Any fx	Ost fx	MOF	Hip fx
Falls at baseline adjusted for FRAX	HK	1.87 (1.34, 2.62)	1.76 (1.20, 2.59)	1.94 (1.28, 2.96)	1.47 (0.78, 2.78)
	SW	1.56 (1.23, 1.97)	1.45 (1.12, 1.88)	1.44 (1.09, 1.90)	1.29 (0.82, 2.01)
	US	1.61 (1.39, 1.88)	1.49 (1.25, 1.78)	1.45 (1.17, 1.80)	1.69 (1.23, 2.31)
	Total	1.63 (1.45, 1.83)	1.51 (1.32, 1.73)	1.51 (1.29, 1.77)	1.54 (1.21, 1.95)
Falls at baseline adjusted for femoral neck BMD	HK	1.92 (1.38, 2.69)	1.82 (1.24, 2.67)	2.00 (1.31, 3.03)	1.68 (0.89, 3.14)
	SW	1.64 (1.29, 2.07)	1.52 (1.17, 1.96)	1.50 (1.14, 1.99)	1.31 (0.84, 2.05)
	US	1.69 (1.45, 1.96)	1.56 (1.31, 1.86)	1.54 (1.24, 1.90)	1.82 (1.33, 2.48)
	Total	1.71 (1.51, 1.92)	1.58 (1.38, 1.81)	1.58 (1.35, 1.85)	1.64 (1.29, 2.08)
High FRAX (MOF with BMD) adjusted for falls	HK	2.41 (1.74, 3.33)	3.00 (2.11, 4.26)	3.15 (2.13, 4.65)	5.13 (2.98, 8.85)
	SW	1.72 (1.36, 2.16)	1.80 (1.41, 2.30)	1.94 (1.49, 2.52)	1.79 (1.19, 2.71)
	US	1.97 (1.70, 2.28)	2.10 (1.78, 2.48)	2.25 (1.85, 2.75)	2.79 (2.08, 3.75)
	Total	1.96 (1.69, 2.27)	2.17 (1.72, 2.74)	2.30 (1.84, 2.88)	2.86 (1.73, 4.75)

Fx = fracture; Ost = osteoporotic; MOF = major osteoporotic fracture; HK = Hong Kong; SW = Sweden; US = United States.

Data are hazard ratios (95% CI) adjusted for age and time since baseline.

### Interactions between past falls, age, follow‐up time, and risk of incident fracture

**Figure 1:**
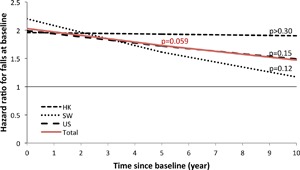
Interaction between past falls and follow‐up time, and risk of any incident fracture.

In both Sweden and the US, there was a tendency for the hazard ratio for fracture associated with past falls to reduce with increasing follow‐up time (*p* interaction = 0.12 and 0.15, respectively). In contrast, no decline with time was observed in the Hong Kong cohort (*p* > 0.30). The interaction between past falls and follow‐up time became close to statistical significance (*p* = 0.059) when all three cohorts were combined (Fig. 1). There was no evidence of an interaction with age. No interactions for either follow‐up time or age with high FRAX probability were observed.

## Discussion

In this large combined population cohort of older men, we have demonstrated that previous falls and high FRAX probability independently predict the risk of future fracture. These findings clearly confirm the value of falls in fracture risk assessment and demonstrate that consideration of past falls yields information over and above that captured by the FRAX algorithm.

The predictive value of past falls for future fracture is well established,^24^ but the present study, to our knowledge, provides the first evidence from a large population‐based cohort that this risk is independent of that captured by FRAX with or without BMD. It complements our previous findings, from the MrOS Sweden cohort, of similar predictive value of past falls and FRAX probability for future falls,^11^ extending this to the key musculoskeletal consequence, namely fracture. Similar to the present study, although risk factors for falls and fracture overlap substantially, and many of which are captured in the FRAX tool, the magnitude of the predictive value of past falls or high FRAX probability was not materially altered by mutual adjustment, indicating that falls history is likely to inform risk not captured by FRAX probability. Interestingly, prior falls predicted incident clinical vertebral fracture as well as the other fracture types. Although vertebral fractures in women have largely been thought to result from actions such as lifting, rather than from falls,^25^ data from the US MrOS cohort suggested that falls were common antecedents of clinical presentation with a vertebral fracture amongst older men.^26^

These findings support the notion that consideration of falls history is likely to add usefully to risk assessment based on the FRAX tool and as such will be of relevance to a large number of guidelines globally.^6^ Although falls have been incorporated into risk calculators derived from single cohorts in which these outcomes have been recorded,^7^, ^8^, ^9^, ^27^, ^28^ the lack of standardized documentation of falls events across the 23 cohorts used in the development and validation of the FRAX tool has meant that the use of prior falls as a clinical risk factor was not possible.^4^ A further consideration is that FRAX input variables were selected on the basis of at least partial independence of BMD and of constituting a risk amenable to pharmacological therapeutic intervention. Although our present findings strongly support the first of these criteria, there is still limited evidence that interventions to reduce falls will also reduce fractures^2^, ^3^, ^4^, ^29^, ^30^, ^31^, ^32^, ^33^, ^34^ or that falls risk is amenable to intervention with pharmacological agents such as bisphosphonates.^4^, ^5^ In one study, baseline risk of falling was not associated with differences in anti‐fracture efficacy of clodronate,^35^ suggesting efficacy in fallers and non‐fallers alike. In contrast, in a trial of risedronate in elderly women selected partly on the basis of high falls risk, the intervention did not lead to statistically significant reductions in fractures.^36^

Recognizing the limitations of falls data in the current FRAX cohorts, a report of an International Society for Clinical Densitometry/International Osteoporosis Foundation Task Force recommended that FRAX probability may be modified to account for a history of prior falls, with the output inflated by 30% (multiplied by 1.3) for each past fall (for up to 5 falls).^4^ This recommendation is based on the univariate hazard ratio (95% CI 1.1, 1.5) for incident hip fracture associated with a past fall, derived from the Study of Osteoporotic Fractures.^37^ Notably, in this cohort the fall‐fracture relationship became statistically nonsignificant after adjustment for poor health and markers of poor mobility; furthermore, this study did not investigate the predictive power of falls independent of other clinical risk factors or BMD. Although the exact approach to incorporation of prior falls into risk assessment remains to be elucidated, our findings inform clinical care, demonstrating that prior falls indicate increased fracture risk over and above that generated by use of other clinical risk factors and BMD in FRAX. Notwithstanding the inconclusive evidence relating falls interventions to fracture reduction, falls risk should clearly be addressed as part of the risk assessment, in addition to measures specifically aimed at improving bone mineral density.

Our findings of potential time interactions for past falls and incident fractures are intriguing and echo our previous observation that the predictive value of past falls for incident falls in the MrOS Sweden cohort also waned with increasing follow‐up time and was greater at younger ages.^11^ Falls‐related risk factors were found to be predictive of fracture risk over a 2‐year period in a recent US study, but because this investigation did not compare the short‐term relationships with those over a longer time period, it is difficult to draw firm conclusions regarding any temporal variation in effect size with regard to falls and follow‐up.^38^ Although it seems intuitively reasonable that falls might mark out particularly unusual individuals relative to the general population at younger ages, where falls overall are less common, it is perhaps counterintuitive that past falls become less predictive of incident falls with time. It is possible that fallers tend to fracture earlier and become less mobile and perhaps less exposed to falls risk and thus to fracture risk with time. For the moment, however, particularly since the association just failed to reach statistical significance and is inconsistent across the three cohorts, this observation remains of interest but requires replication in other populations.

We studied three well‐characterized cohorts drawn from general populations with standardized assessments and prospective recording of fractures. However, there are some limitations that should be considered in the interpretation of our findings.^16^ First, the population studied was male and of a modest age range (64 to 99 years), so limiting generalizability of our findings. Second, the definition of glucocorticoid use differed from those usually specified for incorporation into FRAX. Third, there was no information on causes of secondary osteoporosis, and this variable was therefore set to missing. The effect of these considerations on our findings is uncertain but may have led to an overall underestimation of risk by FRAX. Finally, we did not have information on the severity of a past fall or whether a past fall was associated with injury, so limiting our ability to identify events potentially most likely to be associated with a fracture outcome.

In conclusion, we have demonstrated that prior falls are a risk factor for incident fracture, independently of FRAX probability calculated with or without BMD. Although our findings clearly demonstrate the value of falls history in fracture risk assessment, further prospective studies in cohorts with wider age ranges, other ethnicities, and, most importantly, women are now warranted to replicate and extend these findings, ideally to establish the potential for inclusion of falls as a modifier of FRAX probability.

## Disclosures

All authors state that they have no conflicts of interest.

## Supporting information

Supporting Table S1.
