# Supplementary material for: Falls Predict Fractures Independently of FRAX Probability: A Meta‐Analysis of the Osteoporotic Fractures in Men (MrOS) Study
Source: J Bone Miner Res. 2017 Dec 8;33(3):510–6. doi: 10.1002/jbmr.3331 (PMC5842893; doi:10.1002/jbmr.3331)
Supplement: Supplementary file 1 — Supporting Table S1. [file JBMR-33-510-s001.docx]

**Falls predict fractures independently of FRAX probability: A meta-analysis of the Osteoporotic Fractures in Men (MrOS) Study**

Nicholas C Harvey^1,2^, Anders Odén^3,4^, Eric Orwoll^5^, Jodi Lapidus^6^, Timothy Kwok^7^, Magnus K Karlsson^8^, Björn E Rosengren^8^, Östen Ljunggren^9^, Cyrus Cooper^1,2,10^, Eugene McCloskey^4,11^, John A Kanis^4,12^, Claes Ohlsson^3^, Dan Mellström^3^, Helena Johansson^3,4,12^

**Online Supplementary Material**

**Online Supplementary Table 1:** Hazard ratio (falls vs no falls) for major osteoporotic fracture adjusted for individual FRAX risk factors plus age. All models are adjusted for follow-up time. Data are hazard ratio (falls vs no falls) and 95%CI for merged cohorts (random-effects model).

| **Risk factor** | **Age (years) (HR per year)** | **BMI (HR per 1 kg/m^2^)** | **Previous fracture** | **Family history hip fracture** | **Current smoking** | **Corticosteroid use** | **Rheumatoid arthritis** | **Excess alcohol intake** |
| --- | --- | --- | --- | --- | --- | --- | --- | --- |
| HR (95%CI) | 1.09 (1.08, 1.11) | 0.98 (0.95, 1.01) | 1.67 (1.26, 2.22) | 1.13 (0.94, 1.37) | 1.29 (0.98, 1.71) | 1.26 (0.77, 2.07) | 1.29 (0.61, 2.74) | 0.73 (0.36, 1.46) |

**Online Supplementary Table 2:** Incidence of major osteoporotic fracture according to falls and FRAX status (95% confidence interval).

| **Group** | **Incidence per 1000 person-years (95%CI)** | **HR (95%CI)** |
| --- | --- | --- |
| Low FRAX; no falls (Ref.) | 7.9 (7.2, 8.7) | 1 |
| Low FRAX; prior falls | 13.0 (10.9, 15.3) | 1.66 (1.26. 2.18) |
| High FRAX; no falls | 19.8 (17.2, 22.8) | 2.46 (1.79, 3.38) |
| High FRAX; prior falls | 26.4 (20.8, 33.2) | 3.18 (2.47. 4.08) |

**Online Supplementary Table 3:** Relationships between past falls, FRAX and risk of new OWH or clinical vertebral fracture. Data are hazard ratio (95%CI) adjusted for age and time since baseline.

|  |  | **OWH** | **Clinical vertebral** |
| --- | --- | --- | --- |
| Falls at baseline | Total | 1.59 (1.30, 196 | 1.79 (1.19, 2.69) |
| High FRAX (MOF with BMD) | Total | 1.98 (1.57, 2.50) | 2.30 (1.80, 2.94) |
| Falls at baseline adjusted for FRAX | Total | 1.55 (1.25, 1.90) | 1.73 (1.13, 2.65) |
| Falls at baseline adjusted for femoral neck BMD | Total | 1.60 (1.31, 1.97) | 1.81 (1.19, 2.74) |
| High FRAX (MOF with BMD) adjusted for falls | Total | 1.94 (1.53, 2.47) | 2.26 (1.76, 2.89) |

OWH=osteoporotic fracture without hip fracture (clinical vertebral, humerus, wrist)

**Online Supplementary Table 4:** Relationships between past falls, FRAX and risk of new fracture. Data are hazard ratio (95%CI) adjusted for age and time since baseline. A) BMD T>-2.5; B) BMD T≤-2.5

**A)**

|  |  | BMD T>-2.5 | | BMD T≤-2.5 | |
| --- | --- | --- | --- | --- | --- |
|  |  | **Any fx** | **MOF** | **Any fx** | **MOF** |
| Falls at baseline | Total | 1.66 (1.47, 1.88) | 1.70 (1.26, 2.29) | 1.70 (1.10, 2.61) | 1.21 (0.73, 1.94) |
| High FRAX (MOF with BMD) | Total | 1.87 (1.52, 2.31) | 2.02 (1.65, 2.49) | 1.75 (0.98, 3.10) | 1.74 (0.95, 3.20) |
| Falls at baseline adjusted for FRAX | Total | 1.62 (1.43, 1.84) | 1.56 (1.27, 1.92) | 1.76 (1.14, 2.72) | 1.23 (0.74, 2.04) |
| Falls at baseline adjusted for femoral neck BMD | Total | 1.70 (1.50, 1.92) | 1.63 (1.38, 1.92) | 1.63 (1.06, 2.51) | 1.16 (0.70, 1.91) |
| High FRAX (MOF with BMD) adjusted for falls | Total | 1.83 (1.48, 2.26) | 1.98 (1.61, 2.44) | 1.88 (1.05, 3.36) | 1.81 (0.97, 3.38) |

Fx=fracture; MOF=Major Osteoporotic Fracture

**Online Supplementary Table 5:** Area under the curve (AUC) values for predictive models.

|  |  | **Any fx** | **MOF** |
| --- | --- | --- | --- |
| Falls at baseline | Total | 0.65 | 0.62 |
| High FRAX (MOF with BMD) | Total | 0.69 | 0.72 |
| Falls at baseline adjusted for FRAX | Total | 0.63 | 0.61 |
| Falls at baseline adjusted for femoral neck BMD | Total | 0.65 | 0.63 |
| High FRAX (MOF with BMD) adjusted for falls | Total | 0.69 | 0.72 |

Fx=fracture; MOF=Major Osteoporotic Fracture
